# Editorial: Using virus specific-signatures during infection to characterize host-pathogen interactions

**DOI:** 10.3389/fgene.2023.1290714

**Published:** 2023-09-22

**Authors:** Paula Luize Camargos Fonseca, Rajarshi Kumar Gaur, Eric Roberto Guimarães Rocha Aguiar

**Affiliations:** ^1^ Genetics Department, Biological Sciences Institute, Universidade Federal de Minas Gerais, Belo Horizonte, Brazil; ^2^ Department of Biotechnology, Deen Dayal Upadhyaya Gorakhpur University, Gorakhpur, Uttar Pradesh, India; ^3^ Department of Biological Science, Center of Biotechnology and Genetics, Universidade Estadual de Santa Cruz, Ilhéus, Brazil

**Keywords:** virus-host interaction, molecular signatures, pattern-based identification, nucleic acid patterns, RNA interference

Generally, viruses can be defined as “replicators that encode structural proteins encasing their own genomes” ubiquitous in all biomes (Koonin et al., 2021). However, many viral species do not fit this profile, since they can lack capsids, being considered naked viruses. Additionally, other attributes must be considered when defining a virus: infectivity, parasitism, size, replication machinery and absence of metabolism (Koonin et al., 2021). It has long been proposed that viruses are infectious entities. However, mycoviruses (fungal viruses) are transmitted vertically, as well as other many eukaryotic viruses. Mycoviruses can also be beneficial to their fungal hosts, being classified as commensals or mutualists (Villan Larios et al., 2023). Thus, the term obligate intracellular parasite or infectious is not true for all described viral species. Moreover, viruses were described as small entities visualized only by electron microscopy. However, after the description of giant viruses, this claim was also refuted, since Pandoraviruses are larger than 1uM (Aherfi et al., 2022). Also, Pandoraviruses can encode enzymes involved in the carboxylic acid cycle (Aherfi et al., 2022). Tupanviruses, another group of giant viruses, can encode part of the genes related to the translational machinery, with the exception of the ribosome (Rolland et al., 2020; Aherfi et al., 2022). Although the definition of viruses is not entirely clear, viruses are replicative, fast-evolving, genetically diverse entities that occupy a virtual space that contains all viral groups, even with their differences.

The virosphere is the totality of all viruses and their diversity of functions, morphology and symbiotic interactions with host cells (Koonin et al., 2021). Viruses can present symbiotic relationships with all types of living cells on the globe, animals, plants, fungi, bacteria and protozoa. However, depending on the interaction level or host fitness they can cause pathogenesis instead of providing an adaptive advantage. Recent estimates suggest that the abundance of viral particles is approximately 4.80 x 10^31^, in which the aquatic and soil biomes present the greatest diversity of particles containing 7.32 x 10^28^ and 4.88 x 10^30^, respectively (Cobián Güemes et al., 2016). However, the amount is probable an underestimation of the real abundance. The group of phages (bacterial viruses) is considered the most diverse viral entity on the planet. Different studies often suggest that each bacterium is infected by 10 different phages. If there are 10 million bacteria, it is estimated that there are about 100 million different phages (Rohwer, 2003). Moreover, viruses can be classified into seven different classes according to their genome type: double-stranded DNA (dsDNA), single-sense DNA (ssDNA), dsRNA, (+)RNA, (−)RNA, (+)RNA with reverse transcriptase gene (RT) and dsDNA with an RNA intermediate and RT gene (Koonin et al., 2021). Due to the extensive virosphere dynamics, methods for detection, identification and functional studies are needed to explore the viral realm.

Many methods for viral detection and identification have been proposed and applied in the last years and can be grouped in indirect and direct detection. While indirect methods are carried out by propagating viral particles in cells (*e.g.*, virus isolation methods), direct methods allow the virus to be detected in the initial sample, through identification of the viral genome or associated antibodies (*e.g.*, amplification and antibody-based assays). Direct methods are generally more used in virology (Cassedy et al., 2021). However, these methods allow the detection of previously described viruses and do not explore the diversity of functions that the virosphere can promote in host cells (Cassedy et al., 2021). For example, the gastrointestinal tract (GIT) of ruminants has a high diversity of microorganisms and they play a fundamental role in the digestion of host animals. However, few studies have been developed to investigate the functional role of microorganisms, including viruses, in this environment as highlighted by Rabapane et al. in this Research Topic. Functional genomics studies can be performed more easily through the methodology of High Throughput Sequencing platforms (HTS). HTS allows the detection of known and new viral species and exploration of not only viral diversity but also the role played by these entities in host cells and biomes (Mokili et al., 2012). Several HTS methodologies linked to bioinformatics analysis can be used in this sense: genomics and metagenomics (study of individual and community genomes) and transcriptomics and metatranscriptomics (study of individual and community transcripts).

Genomics can provide essential information about the stability, function, structure and evolution of viral genomes (Casas and Saborido-Rey, 2023). Bacteriophages, in addition to being the most diverse viruses on the planet, also play an essential role in mediating horizontal gene transfer (HGT), mainly related to antimicrobial resistance genes (AMR) (Debroas and Siguret, 2019). Therefore, the sequencing of phage genomes becomes important in the study of the genomic and functional plasticity of this viral group. Taking advantage of phage genome sequencing it was possible to identify the presence of integrons, genetic elements that can serve as gene cassettes in bacterial cells. Moreover, these phage integrons can potentially be used in bacteria and *vice versa*, being a possible route of HGT and be used for bacteria to express and transfer AMR genes (Qi et al.).

HGT events are essential in the development of pathogenic microorganisms. HGT among different realms can occur naturally in ecosystems (Callier, 2019). In the case of viruses, for example, evolution and HGT can drive spillover events from wild hosts to humans. This is due to the domestication of animals, proximity to rural and urban areas and constant changes in the primary viral ecosystems (habitat). The process of alteration of the microbial community may have led to a succession of the community, due to the presence of microorganisms with AMR to biotic and abiotic stresses, which could be passed on to other microorganisms by HGT events carried out by phages and bacteria. During the process of microbial succession, the pathogen can replicate at an increased rates and initiate an infection. Again, viruses may be instrumental in the emergence of bacterial pathogens through HGT events. Apari and Földvári.

Nevertheless, the description of microbiome succession and pathogen spillover is difficult to be studied, since viruses can integrate into the host genome or be integrated into the host genome as an Endogenous Viral Element (EVE) (Aguiar et al., 2020; Veglia et al., 2023). These events alter the expression profile during an interaction and can modulate the response to a viral pathogen. In *Diadegma fenestrale*, the parasitoid wasp, during oviposition, polydnavirus (PDV) are incorporated, as they reside in the ovaries as obligate symbionts. The presence of several EVEs from different PDV viral genes were observed in the host genome, being expressed even for long periods. This study demonstrates viral plasticity and the potential association of co-evolution between virus and host cells and its functional control. (Kim et al.).

Overall, It is well known that the relationship between viruses and their hosts is a co-evolutionary process due viruses require the host`s cellular machinery to translate their proteins. This relationship is under constant adaptation and selective pressures. The host can develop new strategies to fight viral infections, while viruses develop counter-defense measures to maintain the infection (Kaján et al., 2020). Since viruses are subject to the same evolutionary pressures that shape the host genome composition and codon usage, they can share similar characteristics Therefore, hosts and virus coding regions tend to share common features, such as dinucleotide composition and codon usage patterns, which can be used to determine possible virus hosts (Lobo et al., 2009; Aguiar et al., 2016; Fonseca et al., 2020). Another strategy with the potential to determine the possible viral host is the assessment of virus derived small RNAs. Small RNAs are generated by the activation of the RNA interference (RNAi) pathway. RNAi induces silencing of self and non-self RNAs based on sequence similarity and can be used as a hallmark of host antiviral response. This strategy is capable of differentiating between endogenous and exogenous viral sequences and also characterizing the virus genome (Aguiar et al., 2016). Considering the vast availability of public libraries and ongoing viral diversity studies, the number of descriptions of new viral species has increased significantly (Simmonds et al., 2017). Nevertheless, many issues are still found in the identification, characterization of viral genomes and designation of their putative host. These limitations are now starting to be overpassed through the use of host and virus molecular characteristics, such as codon usage, di-nucleotide composition, and virus-derived small RNAs (indicative of RNAi pathways activation), that has helped in the identification and characterization of viral sequences in complex samples and also reveal aspects of the virus-host interactions (Aguiar et al., 2016; Fonseca et al., 2020; Abbo et al., 2023).

We were very happy with the Research Topic “*Using virus specific-signatures during infection to characterize host-pathogen interactions*” and would like to thank all the authors who contributed to their studies. We hope that the research presented on this Research Topic will be helpful to the virus identification and characterization of host-virus interactions.
